# Quantification of Information Exchange in Idealized and Climate System Applications

**DOI:** 10.3390/e21111094

**Published:** 2019-11-08

**Authors:** Praveen Kumar Pothapakula, Cristina Primo, Bodo Ahrens

**Affiliations:** 1 Institute for Atmospheric and Environmental Sciences, Goethe University Frankfurt am Main, Altenhöferallee 1, 60438 Frankfurt am Main, Germany; PrimoRamos@iau.uni-frankfurt.de (C.P.); Bodo.Ahrens@iau.uni-frankfurt.de (B.A.); 2 Senckenberg Biodiversity and Climate Research Centre (SBiK-F), Senckenberganlage, 25, 60325 Frankfurt am Main, Germany

**Keywords:** transfer entropy, information flow, climate informatics, Indian Ocean dipole, El Niño

## Abstract

Often in climate system studies, linear and symmetric statistical measures are applied to quantify interactions among subsystems or variables. However, they do not allow identification of the driving and responding subsystems. Therefore, in this study, we aimed to apply asymmetric measures from information theory: the axiomatically proposed transfer entropy and the first principle-based information flow to detect and quantify climate interactions. As their estimations are challenging, we initially tested nonparametric estimators like transfer entropy (TE)-binning, TE-kernel, and TE k-nearest neighbor and parametric estimators like TE-linear and information flow (IF)-linear with idealized two-dimensional test cases along with their sensitivity on sample size. Thereafter, we experimentally applied these methods to the Lorenz-96 model and to two real climate phenomena, i.e., (1) the Indo-Pacific Ocean coupling and (2) North Atlantic Oscillation (NAO)–European air temperature coupling. As expected, the linear estimators work for linear systems but fail for strongly nonlinear systems. The TE-kernel and TE k-nearest neighbor estimators are reliable for linear and nonlinear systems. Nevertheless, the nonparametric methods are sensitive to parameter selection and sample size. Thus, this work proposes a composite use of the TE-kernel and TE k-nearest neighbor estimators along with parameter testing for consistent results. The revealed information exchange in Lorenz-96 is dominated by the slow subsystem component. For real climate phenomena, expected bidirectional information exchange between the Indian and Pacific SSTs was detected. Furthermore, expected information exchange from NAO to European air temperature was detected, but also unexpected reversal information exchange. The latter might hint to a hidden process driving both the NAO and European temperatures. Hence, the limitations, availability of time series length and the system at hand must be taken into account before drawing any conclusions from TE and IF-linear estimations.

## 1. Introduction

Complex dynamical systems consist of interacting subsystems. It is important to detect these interactions and quantify their strength to improve process understanding. Especially in the climate system, detecting the interactions could provide insights into system dynamics. These interactions can be contemplated as information exchanged or transferred among subsystems. Shannon [1] introduced a mathematical theory for quantifying the information contained in the context of data compression and transmission. Recently, there has been a surge in applications of information theory in a wide range of fields, for example, uncertainty propagation [2,3,4], neurosciences [5,6], climate sciences [7,8,9], earth system sciences [10,11,12,13], turbulence research [14], and networks and synchronization in dynamical systems [15].

Often in climate studies, the relationship among subsystems is assessed with correlation analysis, empirical orthogonal functions, and linear regressions given time series. Among them, correlation analysis is a parametric method used in identifying linear interactions. For nonlinear interactions, mutual information, a nonparametric method [1,16], is often used. Mutual information reveals any shared information between two subsystems. However, both correlation and mutual information are symmetric, i.e., they cannot distinguish between a drive and a response system. For a better understanding of the dynamics, detection of the directionality of interactions is essential. The time-lagged cross-correlation and time-lagged mutual information methods are frequently used for this purpose [17,18]. The authors of [19] observed that these two methods are sensitive to autocorrelations which often obscure detection and quantification of the interaction mechanisms. For example, in his study, a spurious interaction between tropical east Pacific and the northern tropical Atlantic is detected at a time lag 3–6 months while cross-correlations are applied, while the information theoretic-based asymmetric measures detected an interaction between tropical east Pacific and the northern tropical Atlantic at a time lag of 1 month. This interaction time lag is physically consistent with the advection speed of Pacific–Atlantic Walker circulation.

For deeper insights into detecting drive and response linear interactions, the author of [20] proposed a test based on the Wiener principle. According to the Granger test, X causes Y if the past of the system X assists in predicting the future of the system, Y. In a statistical sense, if the error variance of the optimal linear prediction of Y future state based on the past of X and Y has a smaller error variance than considering the past of Y alone, then X causes, Y. A nonparametric method equivalent to Granger causality known as transfer entropy (TE) was proposed by [21] (for linear Gaussian systems, they are equivalent up to a factor of 2). The TE measures if any additional information is provided by the past of the source system, which assists in predicting the future state of the destination system. In other words, it measures the divergence or deviation between the entropy rates of the destination’s own past and the past included from a source system. The TE, unlike mutual information, is an asymmetric quantity and hence, it can detect drive and response interactions. It is worth noting that, in nature, the interactions do not merely consist of driving and responding systems, but also systems which drive each other simultaneously with different interaction strength. Hence, any reliable asymmetric estimate, e.g., TE, should reveal this underlying behavior. Many information-theory-based methods were spawned based on a similar principle to that of TE, for example, momentary information transfer, Information Transfer to Y, and Information transfer to X [22]. These methods have their own applications and limitations.

The simplest estimation of TE uses a multivariate Gaussian model assuming linear interactions between the subsystems. This parametric estimation is hereafter referred to as TE-linear. While the parametric estimator TE-linear is straightforward to calculate, the nonparametric TE estimation is notoriously challenging. Some of the common nonparametric estimation techniques of TE in the literature include the binning, kernel density, and k-nearest neighbor. These estimators are sensitive to the parameter selection in their implementation, such as the bin width selection in the binning estimator, the kernel width in the kernel density estimator, and the number of nearest neighbors while applying the k-nearest neighbor estimator. Unfortunately, no clear consensus is reached among the scientific community on selecting these free parameters. As a result of this dependency, spurious detection of information exchange between the system components could arise. Regardless, TE has been widely used—for example, the authors of [8] applied TE in the identification of primary drivers of recent climate variability and quantified their influence on climate variability. Their results suggested that greenhouse gases are primary contributors to the recent climate variability. The authors of [23] studied the information exchange between the South Atlantic anomaly and global sea level for the last 300 years using TE and concluded that larger information is exchanged from the south Atlantic anomaly to global sea-level rise than vice versa. However, these studies relied only on a single TE estimation technique (binning). It is still unclear if TE nonparametric estimations with the free parameters reliably produce numerically consistent estimations. Furthermore, these estimators are also sensitive to the length of the time series. For example, robust kernel estimation asks for sufficient data. Hence, before applying TE to climate phenomena, we tested various estimators of TE along with their sensitivity on time series length with idealized systems where the system dynamics is expected or known.

Liang and Kleeman [24], realizing that information exchange could be derived rigorously rather than axiomatically, developed another method called it information flow (IF), which is derived from the first principles of information theory. In their framework, the information source and destination are abstracted as system components and thus derive the information flow between these dynamical components. The information flow between the source system Y and destination system X is equal to the difference between the time evolution of marginal entropy of X and entropy of X excluding the influence of, Y. To apply the information flow method, the time evolution of the marginal probabilities must be computed. This time evolution of the marginal probabilities, in turn, depends on the system dynamics. If the system dynamics is unknown, IF becomes difficult to apply. The information flow method was successfully applied to Heńon maps, the Rössler system, and truncated Burgers–Hopf with their respective system dynamics known [25]. Unlike that of IF, the calculation of TE do not require system dynamics. Hence, given two climate time series, TE is straightforward to apply. Liang [26] proposed a simple and concise maximum likelihood estimator of IF for linear systems which is easy and straightforward to apply without system dynamics. This estimator is a very important result for the climate community as it bridges the gap between theory and real-world applications. From hereafter, this maximum likelihood estimator is referred to as information flow-linear (IF-linear). IF-linear has been successfully applied in detecting the causal structure between CO${}_{2}$ and global temperatures [7], changing the relationship between the convection over the Western Tibetan Plateau and the sea surface temperature in the Northern Bay of Bengal [27] and forecasting the tropical cyclone genesis over the Northwest Pacific through identifying the causal factors in cyclone–climate interactions [28].

In this work, our aim was to apply information theory methods to detect interactions between climate phenomena. Moreover, we were provided with a limited amount of temporal series of climate data and with their dynamics unknown. Hence, we focused on IF-linear and TE methods. We wanted to find out if these methods are consistently able to detect the directionality of the interactions for climate phenomena. Having in mind that the nature of both methodologies differs, we first wanted to understand if this could lead to differences in detecting the information exchange from climate time series. On the other hand, TE methods are highly sensitive to the choice of free parameters and with time series length, which often might lead to brittle information exchange detections. Thus, we initially checked if the different estimators (IF-linear, TE-linear, TE-binning, TE-kernel, and TE-kraskov) detect the directionality of the interactions using various temporal series lengths as realized by idealized systems, whose dynamics and directionality of the interactions is expected or known. These systems consist of uni- and bidirectional coupled linear and nonlinear systems. Thereafter, we applied these methods to the Lorenz-96 system [29], which is known to mimic the mid-latitude atmosphere behavior. Finally, we experimentally applied these methods to (1) Indo-Pacific sea surface temperatures interbasin coupling and (2) the relation between North Atlantic Oscillation (NAO) and winter near-surface air temperatures over Europe. One of the limitations of this study is that we rigorously tested and applied various estimators to two-dimensional systems only. For a detailed and excellent review on the applications of TE on high dimensional interactions, refer to [30].

This paper is organized as follows. Section 2 comprises the background material for IF-linear, TE, and its estimation techniques such as TE-linear, TE-binning, TE-kernel, and TE k-nearest neighbor. In Section 3, the abovementioned methods are applied to uni- and bidirectional coupled linear and nonlinear systems, the Lorenz-96 system, and then to climate phenomena. Results are also discussed in this section. Finally, conclusions are drawn in Section 4.

## 2. Methods

In this section, the basic concepts of information theory are discussed along with a brief introduction of information flow and transfer entropy. Estimation techniques of TE are also presented.

### 2.1. Transfer Entropy

Let p(x) be the probability of a state for the random variable *X*. The Shannon entropy of *X*, H(X), quantifies the amount of information needed to describe the random variable *X* [1]:

$$\begin{array}{c} H\left(X\right)=-\sum_{x}p\left(x\right)\log p\left(x\right), \end{array}$$

where the summation goes through all states of the random variable *X*. The units of entropy are nats if a natural logarithm is applied; alternatively, it is often expressed in bits when the logarithm base is 2.

Let q(x) be another probability distribution for the same random variable, and Kullback divergence measures the distance between the two probability distributions *p* and *q*. It is defined as:

$$\begin{array}{c} D_{p||q}=\sum_{x}p\left(x\right)\log\frac{p(x)}{q(x)}. \end{array}$$

Similarly, mutual information(MI) is the divergence between the joint distribution p(x,y) of variable X and Y and the product of the marginal distributions p(x) and p(y):

$$\begin{array}{c} {\mathrm{MI}}_{xy}=\sum_{x,y}p\left(x,y\right)\log\frac{p(x,y)}{p(x)p(y)}. \end{array}$$

Mutual information quantifies the shared information between X and Y and thus is symmetric and lacks any direction of information exchange. Mutual information between X and Y while a third variable (Z) taken into account is given by conditional mutual information (CMI) as:

$$\begin{array}{c} {\mathrm{CMI}}_{xy|z}=\sum_{x,y}p\left(x,y,z\right)\log\frac{p(x|y,z)}{p(x|z)}. \end{array}$$

Transfer entropy is a special case of CMI, in which the conditioning is done on the past of Y inplace of, Z. Furthermore, the TE reflects the dependencies contained in the transitional probabilities which represent the dynamics of the system. The TE measures the deviation between the generalized Markov property, in other words, the deviation between the transitional probabilities $p(x_{n+1}|x_{n},...,x_{n-k+1})$ and $p(x_{n+1}|x_{n},....,x_{n-k+1},y_{n},....,y_{n-l+1})$:

(1) $${\mathrm{TE}}_{y->x}=\sum_{x,y}p\left(x_{n+1},x_{n}^{k},y_{n}^{l}\right)\log\frac{p(x_{n+1}|x_{n}^{k},y_{n}^{l})}{p(x_{n+1}|x_{n}^{k})},$$

where *k* and *l* are the embedding dimensions of the destination and source variables, respectively. An important measure known as active information storage (AI) is given as:

$$\begin{array}{c} \mathrm{AI}=\mathrm{MI}(x_{n}^{k};x_{n+1}) \end{array}$$

which is used in approximating the embedding dimensions of the destination system. It measures how much of the information from the past of the X system $\left(x_{n}^{k}\right)$ is observed to be in use in computing its next future observation $\left(x_{n+1}\right)$. Here, MI refers to mutual information. If additional information from $y_{n}$ assists in the reduction of the uncertainty in the future state of $x_{n+1}$ given $x_{n}$, then there is an information transfer from Y to, X. Thus, TE quantifies the average information transfer from Y to X and similarly vice versa. It is important to note that the source Y can potentially influence the system X at various interaction delays. A scanning approach for the largest information exchange values was employed by [31] to calculate the interaction delay between the source and destination systems. In this work, we adopted the same methodology to calculate the interaction delays for climate applications. Furthermore, the comparison of TE estimations only makes sense if the interaction delays in both directions are properly and independently reconstructed [32]. Hence, it is important to ensure the correct interaction delays are extracted. The TE, unlike MI and correlation, is an asymmetric measure. While the TE equations seem straightforward, its nonparametric estimation for continuous data is quite challenging. Coarse-graining the continuous data into discrete states is hard to interpret unless the measure converges when reducing the coarsening scale. The implementation of the binning estimator uses a bin width parameter upon which the estimates are highly sensitive. Similarly, the kernel density estimator relies on kernel width and the k-nearest neighbor estimator on the number of nearest neighbors. Unfortunately, no clear consensus is reached in optimally choosing these parameters. Hence, in the current study, we tested all the methods for numerical consistency before applying to climate applications. A brief introduction to these methods is given in the following sections.

#### 2.1.1. Estimation of TE-Binning

For the estimation of TE, joint probability distributions are calculated from the underlying time series. Binning is one of the most straightforward approaches in the estimation of joint probabilities. Unfortunately, entropy estimations are highly sensitive to the number of bins chosen (i.e., bin width). In the literature, there exist numerous methods that describe the selection of an optimum number of bins for Gaussian distributions [33,34] and suggestions for distributions without any underlying assumptions [35,36]. In the current study, we used a more general method proposed by [37], known as generalized Knuth method with no underlying assumptions about the distribution. In this method, each of the N observed data points are placed into one of M-fixed width bins, where the number of bins is selected utilizing a Bayesian paradigm. The likelihood of the multidimensional data with volume V and probabilities $\pi_{i}$ for the $i^{th}$ bin is given by the multinomial distribution:

$$\begin{array}{c} p\left(d|M,\pi \right)={\left(\frac{M}{V}\right)}^{N}\pi_{1}^{n_{1}}\pi_{2}^{n_{2}}...\pi_{M}^{n_{M}}, \end{array}$$

where $d=[d_{1},d_{2}...,d_{N}]$ denote N observed points, $n_{1},n_{2}...n_{N}$ denotes the number of points in each sample, and $\pi =[\pi_{1},\pi_{2},..,\pi_{M}]$ denote the respective probabilities of the bin. The Dirichlet prior conjugate to the multinomial likelihood function for the non-informative prior is given as:

$$\begin{array}{c} p\left(\pi |M\right)=\frac{\Gamma \left(\frac{M}{2}\right)}{\Gamma (\frac{1}{2})}{\left[\pi_{1},\pi_{2},...,\pi_{M-1},\left(1-\sum_{i=1}^{M-1}\pi_{i}\right)\right]}^{-1/2}. \end{array}$$

The posterior distribution of the bin probabilites from the Bayes theorem is:

$$\begin{array}{c} p(\pi ,M|d)\propto p(\pi |M)p(M)p(d|\pi ,M). \end{array}$$

Then, the optimum number of bins are chosen by the mode of the posterior distribution of:

$$\begin{array}{c} \log p\left(\frac{M}{d}\right)=N\log M+\log\Gamma \left(\frac{M}{2}\right)-M\log\Gamma \left(\frac{1}{2}\right)-\log\Gamma \left(N+\frac{M}{2}\right)+\sum_{i=1}^{M}\log\Gamma \left(ni+\frac{M}{2}\right)+K, \end{array}$$

where *M* is the number of bins, *d* is the observed data points, ni is the number of data sample in each bin, and *K* is a constant. For more details, refer to [37]. After calculating the optimal number of bins *M*, TE is calculated by decomposing into individual joint entropies given as:

(2) $$\begin{array}{c} \begin{array}{c} {\mathrm{TE}}_{y->x}=H\left(x_{n}^{k},y_{n}^{l}\right)-H\left(x_{n}^{\left(k+1\right)},y_{n}^{l}\right)+H\left(x_{n}^{\left(k+1\right)}\right)-H\left(x_{n}^{k}\right), \end{array} \end{array}$$

where *k* and *l* are the embedding dimensions of X and Y, respectively. An estimation bias for TE could arise due to the assumption of a uniform distribution within every single bin which corresponds to maximum entropy.

#### 2.1.2. Estimation of TE-Kernel

This estimator uses the box step kernel Θ with Θ(x>0)=0 and Θ(x<0)=1 for the estimation of relevent joint probability distributions (e.g., $\hat{p}\left(x,y\right)$, $\hat{p}\left(x\right)$ and $\hat{p}\left(y\right)$). For example, the joint probability distribution $\hat{p}\left(x,y\right)$ is calculated as:

$$\begin{array}{c} {\hat{P}}_{r}\left(x_{n},y_{n}\right)=\frac{1}{N}\sum_{n^{\prime }=1}^{N}\Theta (|\left(x_{n}-x_{n^{\prime }}\right),\left(y_{n}-y_{n^{\prime }}\right)\left|-r\right), \end{array}$$

where the norm corresponds to the maximum distance in the joint space and *r* is the kernel width. In simple terms, the resultant probability is the fraction of N values which fall within the kernel width *r* in the joint dimensional space. Here, *r* is the free parameter and the resultant probability is sensitive to the choice of *r*. Furthermore, the conditional probabilities are defined in terms of their respective component joint probabilities. These probabilities are substituted in Equation (1) to calculate TE. Kernel estimators are model-free (i.e., they do not assume parametric distribution). For more details about the estimator, refer to [38,39] and information-theoretic toolkit from [40].

#### 2.1.3. Estimation of TE-K-Nearest Neighbor

The authors of [41] introduced the k-nearest neighbor estimator which uses an adaptive binning strategy. This estimator uses the average distances to the k-nearest neighbor data points for the calculation of TE. This is a nonparametric estimation technique.

Transfer entropy from Y to X, $TE_{yx}$ with embedding dimensions k=1 and l=1, is calculated as follows: For each point in the highest dimensional space given in Equation (2) (i.e., $z_{i}=\left(x_{n+1},x_{n},y_{n}\right)$), its neighbors’ distance *d*=max$\left|\right|z_{i}-zj\left|\right|$ is calculated. While any norm could be used, in the current study, the maximum norm is utilized. The number of points that fall within the range *d* in all the marginal spaces is counted. Thereafter, the number of points in each of the marginal spaces are substituted in the equation below to calculate TE:

$$\begin{array}{c} {\mathrm{TE}}_{y->x}=\Psi \left(K\right)+<\Psi \left(n_{x_{n}}+1\right)-\Psi \left(n_{x_{n+1}},n_{x_{n}}\right)-\Psi \left(n_{x_{n}},n_{y_{n}}\right)>, \end{array}$$

where K is the number of nearest neighbors, Ψ denotes the digamma function, while the angle brackets indicate averaging over all the points, $n_{x_{n+1}},n_{x_{n}}$ and $n_{y_{n}}$ are the number of points that fall within the range *d* in the marginal spaces. Similarly, this formula can be extended for higher embedding spaces. Furthermore, this method enables for bias correction. From hereafter, this method is referred as TE-kraskov. For more details, refer to the information-theoretic toolkit of [40].

#### 2.1.4. Estimation of TE-Linear

The entropy for a continous random variable *X* is given as:

$$\begin{array}{c} H\left(x\right)=-\int_{-\infty }^{\infty }p\left(x\right)\log p\left(x\right)dx. \end{array}$$

For the linear estimation of entropy, substituting the probability of a Gaussian distribution [42] in the above equation gives:

$$\begin{array}{c} H\left(x\right)=\frac{1}{2}\log2\pi e\sigma^{2}. \end{array}$$

For a multivariate Gaussian model, the entropy is given as:

$$\begin{array}{c} H\left(x\right)=\frac{1}{2}{\log(\left(2\pi e\right)}^{d}|\Omega_{x}\left|\right), \end{array}$$

where *d* is the number of dimensions, $|\Omega_{x}|$ is the determinant of the d×d covariance matrix $\Omega_{x}={\bar{xx}}^{T}$, and the overbar indicates averaging. Furthermore, the TE is estimated as the sums and differences of the joint entropies given in Equation (2).

#### 2.1.5. Assumptions in the Practical Estimation of TE

Before applying the TE estimators mentioned above, it is very important to observe their assumptions. While the TE estimations in the above sections involve the interactions between two subsystems X and Y, real-world climate applications often involve a tangle of higher dimensional interactions. In order to disentangle the sole interactions between X and Y, one needs to remove the influence of all the other interacting variables in the state phase. However, this can be computationally exhaustive and perhaps almost impossible. This unreal aspect was already mentioned in [20]. Hence, while applying TE to retrieve the information exchange between two subsystems, there might be unobserved variables influencing the estimation. Hence, this limitation needs to be accounted for while applying TE. In the current study, we limited our detailed investigations to two-dimensional systems. Hence, a possible influence of unobserved variables on TE estimations cannot be ruled out. For a detailed review on the applications of TE on high dimensional interactions, refer to [30].

In the TE Equation (1), the deviation between the transitional probabilities is calculated with the embedding dimensions of the destination and source variables, *k* and *l*, respectively. The embedding dimension *l* of the source variable could be chosen at which the maximum information exchange takes place, while an ideal embedding *k* for the destination system should be as large as possible. Due to the computational complexity and a limited number of time series data available for climate applications, a minimum value for k=1 could be chosen provided most of the information from the past of destination system is embedded within the minimum *k*. In the current study, this was verified through the calculation of active information of the destination system. Moreover, assumptions in the TE calculation involve stationarity, time aggregation, faithfulness, etc. An excellent review of these assumptions is available in [30].

### 2.2. Liang and Kleeman Information Flow

Consider a two-dimensional nonlinear system $x_{1}$ and $x_{2}$:

$$\begin{array}{c} \frac{dx_{1}}{dt}=F_{1}\left(x_{1},x_{2},t\right),\\ \frac{dx_{2}}{dt}=F_{2}\left(x_{1},x_{2},t\right), \end{array}$$

with randomness limited to its intial condition. The above equations follow the convention in physics which does not distinguish random and deterministic variables. Let $H_{1}$ be the marginal entropy of $x_{1}$. The $H_{1}$ evolution of $x_{1}$ may be due internal mechanism of $x_{1}$ itself or subject to the influence of $x_{2}$. The later is the information flow from $x_{2}$ to $x_{1}$, which is of interest to us . If $\frac{dH_{1}^{*}}{dt}$ is the contribution of evolution of marginal entropy by $x_{1}$ itself, then:

$$\begin{array}{c} \frac{dH_{1}}{dt}=\frac{dH_{1}^{*}}{dt}+T_{2->1}, \end{array}$$

where $T_{2->1}$ is the information flow from $x_{2}$ to $x_{1}$. Therefore, the information flow could be written as:

$$\begin{array}{c} T_{2->1}=\frac{dH_{1}}{dt}-\frac{dH_{1}^{*}}{dt}. \end{array}$$

The term $\frac{dH_{1}}{dt}$ can be calculated by Liouville equation. Thus, Liang and Kleeman [24] obtained the entropy evolution: $\frac{dH}{dt}=E\left(\nabla \cdot F\right)$, where *E* is the expectation operator. Based on this argument, the authors of [24] argued that the first term on the right hand side must be $\frac{dH_{1}^{*}}{dt}=E\left(\frac{\partial F_{1}}{\partial x_{1}}\right)$, which was later proven by [25]. Substituting both terms in the above equation, the authors of [24] argued that the information flow from $x_{2}$ to $x_{1}$ is equal to:

$$\begin{array}{c} T_{2->1}=\frac{dH_{1}}{dt}-\frac{dH_{1}^{*}}{dt}=-E\left(\frac{1}{\rho_{1}}\frac{\partial F_{1}\rho_{1}}{\partial x_{1}}\right), \end{array}$$

where $\rho_{1}$ is the marginal probability density function of $x_{1}$. However, this heuristic argument was rigorously proven in [43,44]. The thus obtained information flow is asymmetric between $x_{1}$ and $x_{2}$. However, the above formalism is only for 2D deterministic systems. Moreover, for a 2D system with stochasticity involved, this formalism does not work. The Liang information flow rigorous formalism has undergone recent developments with higher dimensional systems and stochasticity involved. Consider a dynamical system:

$$\begin{array}{c} \frac{dx}{dt}=F\left(t;x\right)+B\left(t;x\right)\dot{w}, \end{array}$$

where x and F are *n*-dimensional vector, B is an n×m matrix, and w is an *m*-vector of standard Wiener process ($\dot{w}$ is a vector of white noise). The rate of information flow from $x_{2}$ to $x_{1}$ for the above dynamical system is given by:

$$T_{2->1}=-\int_{R_{n}}\rho_{2/1}\frac{\partial (F_{1}\rho_{2})}{\partial x_{1}}dx+\frac{1}{2}\int_{R_{n}}\rho_{2/1}\frac{\partial^{2}\left(g_{11}\rho_{2}\right)}{\partial x_{1}^{2}}dx,$$

where $\rho_{2/1}$ is the conditional probability density function of $x_{2}$ on $x_{1}$, $\rho_{2}=\int_{R}\rho dx_{2}$ and $g_{11}=\sum_{j=1}^{m}b_{1j}b_{1j}$. When n=2, the equation reduces to:

$$T_{2->1}=-E\left[\frac{1}{\rho_{1}}\frac{\partial (F_{1}\rho_{1})}{\partial x_{1}}\right]+\frac{1}{2}E\left[\frac{1}{\rho_{1}}\frac{\partial^{2}g_{11}\rho_{1}}{\partial x_{1}^{2}}\right].$$

If the system dynamics $F_{1}$ and $g_{11}$ are independent of $x_{2}$, then $T_{2->1}=0$, which remarkably appears in the classical formalism. For systems with the dynamics unknown, the estimation of entropy evolution is a challenge. Hence, Liang [26], under the linear assumption, proposed a simple easy-to-use formula know as the maximum likelihood estimator of information flow. Given two series $x_{1}$ and $x_{2}$, for consistency with the formulae of TE mentioned above, we considered $x=x_{1}$ and $y=x_{2}$; the information flow maximum likelihood estimator or IF-linear from the system *y* to *x* is given by:

$$\begin{array}{c} T_{y->x}=\frac{C_{xx}C_{xy}C_{y,dx}-C_{xy}^{2}C_{x,dx}}{C_{xx}^{2}C_{yy}-C_{xx}C_{xy}^{2}}, \end{array}$$

where $C_{xx},C_{yy}$ and $C_{xy}$ are the covariances of *x* and *y*, while the subscript dx indicates time series derived from *x* which is formed as $\frac{x(n+k)-x(n)}{k.dt}$, with k some integers greater than or equal to 1. This easy-to-use formula bridges the gap between theory and real applications and has been successfully applied to real-world applications.

## 3. Results

In the current section, the above-discussed methods are tested for one-way and two-way coupled linear and nonlinear idealized systems. After testing various estimators, we applied them to the Lorenz-96 model which mimics midlatitude atmosphere behavior and finally to two important real-world climate phenomena.

### 3.1. Applications to Idealized Systems

#### 3.1.1. Unidirectional Linearly-Coupled Autoregressive System

We considered a two-dimensional linear system *x* and *y* with the following governing [37] :

(3) $$\begin{array}{c} \begin{array}{c} \begin{array}{cc} y_{n+1} & =0.5y_{n}+N\left(0,1\right),\\ x_{n+1} & =0.6x_{n}+Cy_{n}+N\left(0,1\right), \end{array} \end{array} \end{array}$$

where N(0,1) is Gaussian noise with zero mean and unit variance. The coupling coefficient *C* is varied from 0 to 1 with an increment of 0.1. The system was initialized with $\left(x_{0},y_{0}\right)=\left(0,0\right)$. We integrated around 100,000 iterations and considered the last 5000 steps for detecting and quantifying the information exchange. Throughout this study, in all the idealized systems, a similar number of iterations was followed. In real climate applications, the number of observations is limited and it is essential that the methods mentioned in Section 2 detect the direction of information exchange even with a limited number of data points. Hence, the total number of points was decreased from 5000 down to 200 time units to check the reliability of these methods. It is worth noting that this article does not aim at suggesting solutions for finite sample size effects; rather, we compared different estimators robustness against variations of the sample size. Equation (3) implies that the coupling coefficient *C* drives the information exchange from *y* to *x*. However, there is no exchange from *x* to *y*. Thus, any reliable method should reproduce this asymmetry. We also show the error bars representing two standard deviations representing the measure of uncertainty for the IF-linear and TE estimations. Furthermore, in order to choose the embedding dimensions for TE, we calculated the active information for the system *x* and *y*. For the source embedding dimensions for TE calculations, we chose l=1, as the governing equations showed the past of source variable exchanges maximum information at time lag 1. The active information for the destination systems showed a minimal increase of AI from 0.2270 nats with time lag 1 to 0.2278 nats with 1 to 10 time lags. Hence, to reduce the computational complexity we chose the value of embedding dimension *k* to be 1. From the governing equations, the interaction time delay of 1 is chosen as the source transfers maximum information exchange at a time delay of 1.

Before applying the TE-binning estimator, Knuth’s method for detecting the optimal number of bins was applied to the data. However, as the data consist of various coupling strengths (C), the optimal number of bins varies for a particular coupling coefficient. For consistency, we kept the bin width constant throughout all the values of coupling coefficient *C* but allowed variations for different length of time series.

**Figure 1:**
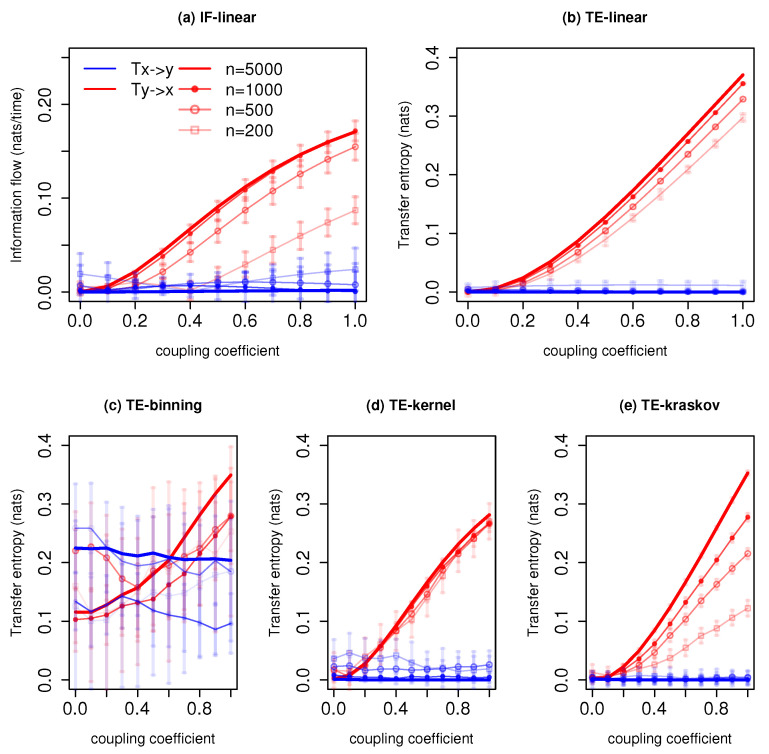
Information exchange in the unidirectional coupled linear system (Equation (3)) with various time series lengths (*n*) measured by (**a**) the IF-linear method (nats/time) and (**b**–**e**) with different variants of the TE measure (in nats). Error bars represent two standard deviations of the permuted surrogate samples.

Figure 1 shows the information exchange from *x* to *y* for different coupling coefficients *C* and varying time series lengths. The IF-linear robustly measures a nonzero information exchange from *y* to *x* and zero information exchange from *x* to *y* for time series lengths n≳500 time units. As expected from the governing equations, the information exchange increases from *y* to *x* with the coupling strength [37]. We also plotted the error bars which represent two standard deviations of IF-linear estimation for the respective time series length (see [26] for details of the significance test). With time series of 200 time units, relying on the error bars, one can distinguish the asymmetry in the information exchange between systems *x* and *y*. The TE-linear estimator also shows the asymmetry in the interactions between *x* and *y*. The results are also stable with all the time series lengths. The error bar represents two standard deviations of 100 permuted surrogates for TE estimations (refer to [40] for details into the significance test for TE). As in the previous case, the strength of the association from *y* to *x* also increases with the increase in the coupling coefficient.

Among the nonparametric estimators, TE-binning is able to reproduce the information exchange from *y* to *x* with a time length n=5000 and n=3000 time units (with a total number of 12 and 14 bins, respectively). However, it overestimates the TE from *x* to *y*. This overestimation may be attributed to the rough estimation of the joint pdf. Moreover, the assumption of a uniform bin width corresponds to biased entropy estimation. With the large overlapping error bars, it is difficult to distinguish the information exchange direction. The TE-kernel estimator (kernel width = 1) is also able to detect the asymmetry in the coupling between *x* and *y* and also the strength of the association. However, with a time series length n≲500 time units, spurious information exchange is detected from *x* to *y*. Nevertheless, the error bars from *x* to *y* with time series length n≲500 time units cross zero nats, and hence, no significant information exchange takes place. Furthermore, various kernel widths from 0.5 to 2 were tested, and the results are consistent between the kernel widths from 1 to 2. The better performance of TE-kernel might be attributed to the smoother estimation of the pdf when compared with the TE-binning. Similarly, the TE-kraskov estimator (20 nearest neighbors) is able to show the one-way coupling and also the strength of the coupling with the time series n≳500 time units. Below 500 time units, permutation surrogate improves to access the direction of information exchange. The TE-kraskov estimator ess tested between 4 to 60 neighbors, and the results are consistent from 20 to 60 nearest neighbors.

The estimations of information exchange are sensitive to the autocorrelation of the time series [30]. Hence, we calculated and compared the autocorrelations for various systems. For this system, the time lag-1 autocorrelation of the system *x* was around 0.5, and the autocorrelation of the system *y* was around 0.7. The presented results show that both parametric methods provide robust results along with the nonparametric TE-kernel and TE-kraskov estimators above the time series length of 500 time units. In addition, with permutation surrogates, even with time series length of 200 time units, the directionality of the information exchange is retrieved. The TE-binning method produced unreliable results.

#### 3.1.2. Bidirectional Coupled Linear Autoregressive System

Often in climate systems, subsystems mutually exchange information. However, sometimes the interaction strength from one system might be stronger than the interaction in the opposite direction. Hence, it is very important that the methods mentioned in Section 2 not only detect the interactions but also quantify their relative strength. Therefore, as an example, we considered the system with governing equations:

(4) $$\begin{array}{c} \begin{array}{c} \begin{array}{cc} x_{n+1} & =0.1x_{n}+C_{yx}y_{n}+N\left(0,1\right),\\ y_{n+1} & =C_{xy}x_{n}+0.1y_{n}+N\left(0,1\right), \end{array} \end{array} \end{array}$$

where N(0,1) is Gaussian noise with zero mean and unit variance and $C_{yx}$∈[0,1] and $C_{xy}$ =$\frac{1}{2}$$C_{yx}$. We initialized the system with $\left(x_{0},y_{0}\right)=\left(0,0\right)$. From the governing equations, a bidirectional information exchange exists and the relative information exchange from *y* to *x* is stronger than from *x* to *y*, since $C_{yx}$ = 2$C_{xy}$. For the TE estimations, the AI values of this system are similar to those of the unidirectional coupled autoregressive system. Hence, for this system, we considered the embedding dimensions *k* and *l* to be 1. From the governing equations, the interaction time delay of 1 was chosen as the source transfers maximum information exchange at a time delay of 1.

**Figure 2:**
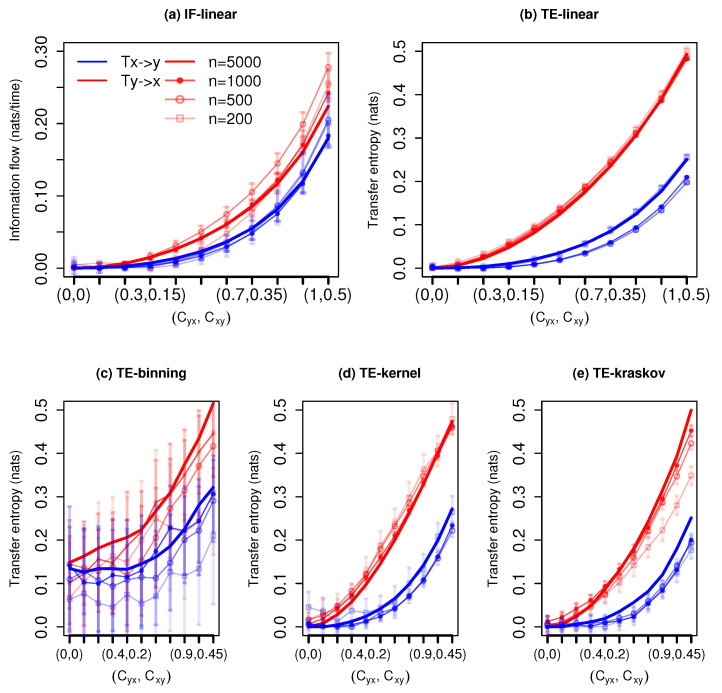
Information exchange in the bidirectional coupled linear system (Equation (4)) with various time series lengths (*n*) measured by (**a**) the IF-linear method (nats/time) and (**b**–**e**) with different variants of the TE measure (in nats). Error bars represent two standard deviations of the permuted surrogate samples.

The measured information exchange with different estimators for the system with governing Equation (4) is shown in Figure 2. The IF-linear shows a bidirectional information exchange. Further, it is able to quantify the strength of the information exchange between *x* and *y* realistically for all time series length; in other words, as the coupling coefficients increases, the information exchange increases bidirectionally, and importantly, the strength of association from *y* to *x* is stronger than vice versa. This measured property would be very useful in climate applications to accurately quantify the strength of the associations among the subsystems. The TE-linear reproduced the bidirectional information exchange for all the time series lengths as well. Further, the strength of the information exchange is also accurately captured.

The TE-binning estimator is able to reproduce the bidirectional information exchange with a time length of n=5000 and n=3000 time units (with a total number of 10 and 13 bins, respectively). However, with time series length less than 1000 time units, spurious detections are noted. Furthermore, with overlapping error bars, the detection of the directionality of information exchange from *y* to *x* and from *x* to *y* is difficult. The TE kernel estimator provides robust bidirectional information exchange (kernel width = 1) but shows spurious results for time series length of 200 time units, especially for weaker couplings. However, at weaker couplings, the error bars assist in detecting the direction of information exchange. Similarly, the TE-kraskov (10 nearest neighbors) estimator is able to show the bidirectional coupling and also the strength of the information exchange for all time series lengths. The results are consistent between 10 to 60 nearest neighbors.

In summary, both the parametric methods produced stable results and also the nonparametric TE-kernel and TE-kraskov estimators showed consistent results for almost all time series lengths. The autocorrelations for this system were 0.2 and 0.4 with time lags of 1 and 2, respectively, for *x* and *y*. Unlike the previous example with unidirectional coupling, the results were relatively robust even with 200 points. This might be associated with the weaker autocorrelation magnitude in the bidirectional coupled system as compared to the unidirectional coupled linear system. This sensitivity of TE estimations on the autocorrelations was reported in [30].

#### 3.1.3. Nonlinear Unidirectional Coupled Anticipatory System

Often in climate science, the relation among the subsystems is nonlinear, and it is very important to identify these associations and quantify their strength. Hence, in this section, we considered a special case of a nonlinear system called anticipatory system. Systems in which the response somehow predicts or anticipates the drive dynamics are known as anticipatory systems [45,46]. For example, the authors of [47] discussed the climate change paradigm illustrating on a systemic framework grounded in the concept of anticipatory system in which the anthroposphere acts as an anticipatory system anticipating and governing the climate dynamics. In the current study, we considered a one way coupled nonlinear anticipation system proposed by [48] with the following equations:

(5) $$\begin{array}{c} \begin{array}{c} \begin{array}{cc} x_{n+1} & =f(x_{n}),\\ y_{n+1} & =\left(1-\epsilon \right)f\left(y_{n}\right)+\epsilon g_{\alpha }\left(x_{n}\right), \end{array} \end{array} \end{array}$$

where f(x)=4x(1−x) is the chaotic logistic map and $g_{\alpha }\left(x_{n}\right)=\left(1-\alpha \right)f\left(x\right)+\alpha f\left(f\left(x\right)\right)$. For this system, ϵ=0.3 is chosen. For ϵ>0.3, the system is synchronized. The response *y* is strongly coupled to the driver, but it retains its independence. The function $g_{\alpha }\left(x_{n}\right)=\left(1-\alpha \right)f\left(x\right)+\alpha f\left(f\left(x\right)\right)$ includes a tunable parameter α (coupling coefficient). When α = 0, the *y* system is driven towards the *x* system and when α=1, the *y* system is driven towards the future of the system *x*. We initialized the system with $\left(x_{0},y_{0}\right)=\left(0.4,0.1\right)$. For the TE calculations, the source embedding dimension l=1 is chosen based on the governing equations, while the AI for the destination system shows a maximum value at time lag 1 and then a decrement for higher lags. Hence, *k* is chosen to be 1. From the governing equations, the interaction time delay of 1 is chosen as the source transfers maximum information exchange at a time delay of 1.

**Figure 3:**
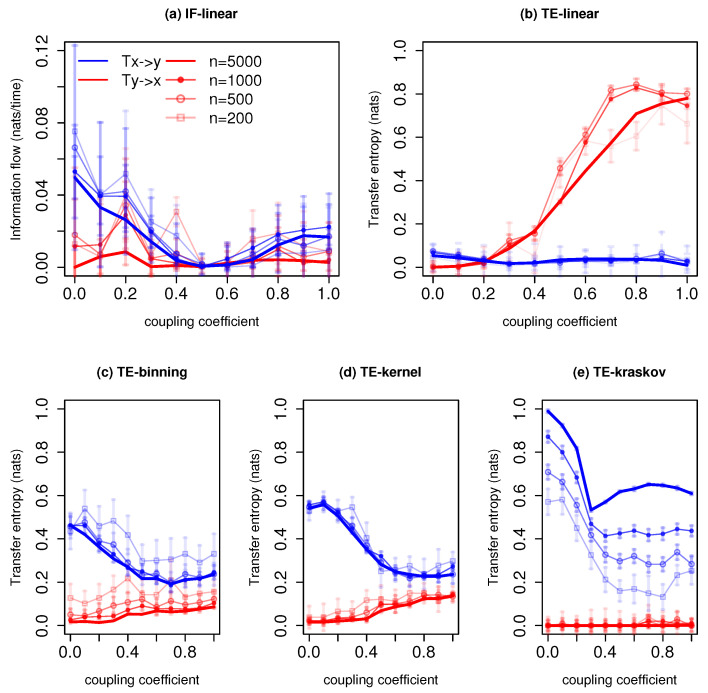
Information exchange in the unidirectional coupled nonlinear anticipatory system (Equation (5)) with various time series lengths (*n*) measured by (**a**) the IF-linear method (nats/time) and (**b**–**e**) with different variants of the TE measure (in nats). Error bars represent two standard deviations of the permuted surrogate samples.

Figure 3 represents information exchange for the system with governing Equation (5). The IF-linear shows an information exchange from *x* to *y* at all coupling coefficients except for the values 0.4 to 0.7 and no information exchange from *y* to *x* except at coupling coefficient 0.2 for the time series length of 5000 time units. Similarly, at lower time series lengths, this asymmetry is captured; however, at coupling coefficients values from 0.4 to 0.7, it is difficult to distinguish the information flow directionality even with the error bars. With longer time series, IF-linear successfully detects the unidirectional information exchange, and this result is reported in [26]. Even though one can argue that IF-linear has been developed strictly for linear systems, it qualitatively detects the asymmetry in the information exchange for this anticipatory system. Liang [26] presented a detailed description of how IF-linear is able to retrieve the properties of this anticipation system through linearization. This property of IF-linear is remarkable, as it is easy to apply given two time series. Moreover, unlike nonparametric TE, IF-linear does not depend on free tuning parameters, which is an added value for its application and also computationally efficient. The TE-linear fails to detect the direction of information exchange at all time series lengths for this system, in fact, the directionality is reversed. It is worth noting that the implementation of TE-linear does not involve any linearization, unlike IF-linear.

The TE-binning estimator (10 bins) shows information exchange from *x* to *y* at a time series length of 5000 time units. At higher coupling coefficients, nonzero information exchange is detected from *y* to *x*. With a time series length less than 1000 time units, unrealistic nonzero information exchange is detected from *y* to *x* at all coupling coefficients. The TE-kernel estimator (kernel width = 0.5) also detects the one-way coupling between *x* and *y* (consistently between 0.25 to 1 kernel widths) at all time series lengths. However, at greater coupling coefficients, spurious information exchange is detected from *y* to *x* at all-time series lengths with kernel width greater than 0.5. The TE-kraskov estimator (4 nearest neighbors) shows exactly the unidirectional coupling between *x* to *y* (consistently between 4 to 60 neighbors) at all time series lengths. The better estimation of TE-kraskov might be attributed to the adaptive data efficient discretization as well as bias correction [41]. However, with TE-kraskov, a very slow convergence of information exchange with increasing time series length is noted. This behavior was reported in the study by [49]. In summary, for this system, IF-linear is able to detect the asymmetry in the information exchange while TE-linear fails. The nonparametric TE-kraskov estimator provides reliable results for all time series lengths. While the asymmetry in coupling is captured by TE-kernel and TE-binning, unrealistic nonzero information exchange is detected at higher coupling coefficients from system *y* to *x*.

#### 3.1.4. Bidirectional Coupled Non-Linear System

In this section, we extend our analysis from a unidirectional nonlinear coupled system to a bidirectionally coupled nonlinear system. For this purpose, we considered Heńon maps which were motivated by the Lorenz equations. The Heńon map captures the stretching and folding dynamics of chaotic systems such as the Lorenz system which mimic the atmospheric behavior. We considered two identically coupled Heńon maps with the following governing equations [50]:

(6) $$\begin{array}{c} \begin{array}{c} \begin{array}{cc} x_{n+1} & =1.4-x_{n}^{2}+0.3x_{n-1}+C_{yx}\left(x_{n}^{2}-y_{n}^{2}\right),\\ y_{n+1} & =1.4-y_{n}^{2}+0.3y_{n-1}+C_{xy}\left(y_{n}^{2}-x_{n}^{2}\right), \end{array} \end{array} \end{array}$$

where the coupling coefficients $C_{yx}$ and $C_{xy}$∈[0,0.4]. For the TE calculations, the source embedding dimension l=1 was chosen based on the governing equations. The AI for the destination system shows a maximum value at first two time lags and then a decrement for higher time lags. Hence, we considered the embedding dimension k=1. From the governing equations, the interaction time delay of 1 was chosen as the source transfers maximum information exchange at a time delay of 1. For this system, the region outside the coupling interval [0.3,0.3] corresponds to synchronization.

**Figure 4:**
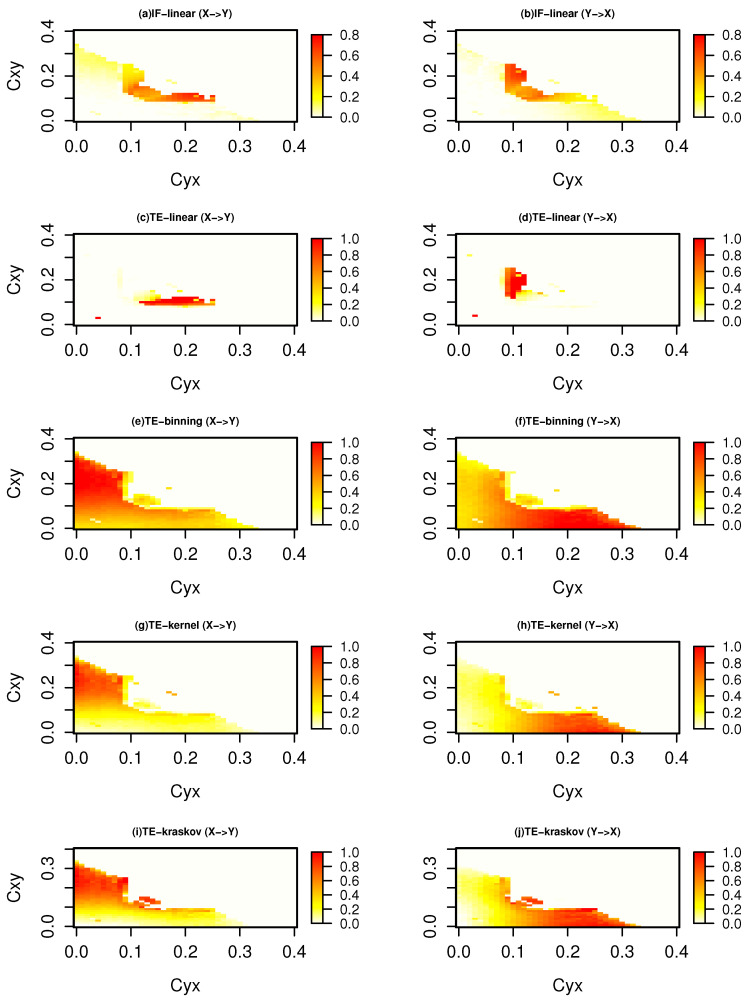
Information exchange in the bidirectional coupled nonlinear system (Equation (6)) with time series length of 500 time units measured by (**a**,**b**) the IF-linear method (nats/time) and (**c**–**j**) with different variants of the TE measure (in nats).

Figure 4 represents information exchange for the system with governing Equation (6) for a time length of 5000 time units. The IF-linear shows an information exchange from *x* to *y* at coupling coefficient $C_{yx}$ from 0.1 to 0.25 and $C_{xy}$ from 0.1 to 0.2. Further, it shows an information exchange from system *y* to *x* at coupling coefficient $C_{yx}$ from 0.1 to 0.3 and $C_{xy}$ from 0.1 to 0.2. A similar behavior was also observed with the TE-linear estimator, but at a lower coupling coefficient below the synchronization, TE-linear fails. The nonparametric TE-binning (consistently between 10 to 20 bins) and TE-kernel (consistently between 0.25 to 1 kernel widths) shows an information exchange between *x* and *y* with couplings below [0.3,0.3]. Furthermore, the strength of the information exchange increases from *x* to *y* as the coupling strength $C_{xy}$ increases and vice versa. This symmetric behavior is expected as the two Heńon systems are identically coupled. This was also shown by [51]. Similar patterns with the TE-kraskov estimator (4 nearest neighbors) are also seen (consistently between 4 to 60 neighbors). We also tested the information exchange for this system with a time series length of 500 time units (figure not shown). It was observed that the spatial patterns of the information exchange exhibit similar spatial patterns as those in Figure 4 but less clearly established for TE-binning, TE-kernel, and TE-kraskov estimators. However, the TE-binning estimation overestimates the information exchange when compared with TE-kernel and TE-kraskov. In summary, for this system, both the parametric methods failed to detect the bidirectional nonlinear interactions, while the nonparametric TE-kernel and TE-kraskov estimators showed consistent results for time series lengths n=5000 and n=500 time units.

From the above-discussed idealized systems, considering the dependencies of TE nonparametric estimations on the free parameters, instead of relying on any single estimator, we propose to use a composite of TE-kernel and TE-kraskov estimators for nonlinear systems. Furthermore, the free parameters, i.e., kernel width and the number of nearest neighbors are to be tuned until both the estimators consistently show a significant information exchange. For the linear systems in addition to TE-kernel and TE-kraskov along with the tuning of free parameters, linear estimators (IF-linear and TE-linear) shall be used simultaneously.

#### 3.1.5. Two-Scale Lorenz-96 Model

So far, we had investigated information exchange in idealized linear and nonlinear coupled systems where the dynamics of the system is known or expected. In this section, we consider a simple conceptual model of atmosphere-like multiscale dynamics, namely, the Lorenz-96 system [29] which consists of coupled fast and slow subsystems. This model was originally introduced to mimic multiscale midlatitude weather. Furthermore, it has been extensively used in the study of the influence of multiple spatiotemporal scales on the predictability of atmospheric flows [3,52]. Although the equations in Lorenz-96 are known and identically coupled, the interaction behavior of Lorenz-96 is hard to expect, as its dynamics is dominated by the interplay between the fast and slow subsystems. Hence, to detect the direction of information exchange, we applied the methods discussed in Section 2 to the Lorenz-96 system. The Lorenz-96 system has the following governing equations:

(7) $$\begin{array}{c} \begin{array}{c} \begin{array}{cc} \frac{dx_{i}}{dt} & =x_{i-1}\left(x_{i+1}-x_{i-2}\right)-x_{i}+F-\frac{hc}{b}\sum_{j=1}^{n}y_{j,i},\\ \frac{dy_{j,i}}{dt} & =cby_{j+1,i}\left(y_{j-1,i}-y_{j+2,i}\right)-cy_{j,i}+\frac{hc}{b}x_{i}. \end{array} \end{array} \end{array}$$

It consists of *m* slow variables $x_{i}$ coupled to m×n fast variables $y_{j,i}$. Furthermore, the two systems are coupled by coupling constant *h*, scaling constants *b* and *c*, and *F* is a constant forcing. Here, i=1,....m and j=1,....n. Both the $x_{i}$ and $y_{j,i}$ have periodic boundary conditions, i.e., $x_{m+1}=x_{1}$; $x_{0}=x_{m}$ and $y_{n+1,i}=y_{1,i+1}$; $y_{0,i}=y_{n,i-1}$. The conventional parameter values F=8 and m=40 are chosen. Furthermore, the values of n,b, and *c* are chosen to be 4, 10, and 10, respectively. This setup leads to a two-scale model where the fast variables fluctuate 10 times faster than the slow ones. The coupling parameter *h* is varied from 0 to 1 with an increment of 0.1. For more details of the system, refer to [52]. For this system, we chose embedding dimensions l=m=1 based on the peak AI values at time lag 1 for TE calculation. From the governing equations, the interaction time delay of 1 is chosen as the source transfers maximum information exchange at a time delay of 1.

**Figure 5:**
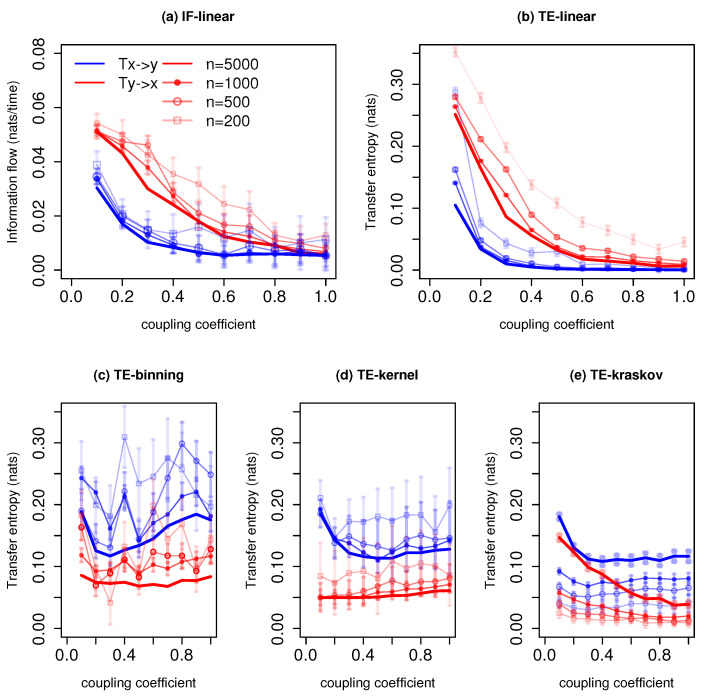
Information exchange in the Lorenz-96 system (Equation (7)) with various time series lengths (*n*) measured by (**a**,**b**) the IF-linear method (nats/time) and (**c**–**e**) with different variants of the transfer entropy (TE) measure (in nats). Error bars represent two standard deviations of the permuted surrogate samples.

Figure 5 represents information exchange for the system with governing Equation (7) for various time series lengths. The IF-linear indicates that there exists a bidirectional information exchange between the slow system and the fast system. Furthermore, the magnitude of information exchange from the fast system to the slow system is stronger than vice versa. The TE-linear shows that a greater amount of information is exchanged from the fast system to the slow system at low couplings. At higher couplings, the information converges to zero in both directions. However, as the IF-linear and TE-linear fail for strong nonlinear systems, we do not draw any conclusions from these results as the Lorenz-96 is a highly nonlinear system.

The TE-binning estimation (10 bins) shows information exchange from *x* to *y*, i.e., the slow system to the fast system. Moreover, weak information exchange is detected from *y* to *x*. The TE-kernel estimator (kernel width = 0.5) and TE-kraskov estimator (4 nearest neighbors) also show that the information exchange is dominant from the slow system to the fast system than vice versa. From the composite use of TE-kraskov and TE-kernel along with the parameter tuning, we see a bidirectional information exchange between the systems *x* and *y*. Moreover, the system *x* leads the system *y*, i.e., the slow system leads the fast system. The authors of [52], in their study, also noted that there exists a bidirectional influence between the fast and slow system as revealed from the spatial patterns of the system trajectories. They also found that the fast system is conditioned by the slow system especially at lower couplings, i.e., the system dynamics is dominated by the slow system. From Figure 5, the nonlinear methods show that the information exchange decreases as the coupling coefficient increases; this requires a further detailed investigation.

### 3.2. Application to Climate Phenomena

#### 3.2.1. Information Exchange between Indian and Pacific Ocean

The El Niño Southern Oscillation (ENSO) is an important large-scale coupled atmosphere–ocean phenomenon which has a remote influence on sea surface temperatures in other ocean basins [17]. An atmospheric bridge is one of the mechanisms through which this influence is mediated [53,54]. ENSO oscillations are characterized by changes in the sea surface temperature (SST) patterns over the equatorial Pacific Ocean. Among many measures, the Niño 4 and Niño 3 indices measure the ENSO oscillation strength and phase. Another important climate variability called the Indian Ocean dipole (IOD) occurs in the Indian Ocean and is known to have an influence on Indian summer monsoon and on rainfall over Australia, Indonesia, and East Africa [53,55,56,57]. A positive phase of IOD represents a cooling in the eastern part of the Indian Ocean and warming in the western part of the Indian Ocean and vice versa. The strength and phase of the IOD are measured through the IOD index.

Earlier studies stated that IOD might arise due to the internal atmosphere–ocean coupling of the Indian Ocean [58]. However, the ENSO forcing is one of the factors responsible for causing IOD, and furthermore, a co-evolution of ENSO and IOD events are noted [59,60,61,62]. ENSO and IOD are known to interact with each other through the Walker circulation in the atmosphere [63]. It is also observed that the IOD could also contribute to the development phase of ENSO through feedbacks [63,64]. Although the ENSO and IOD co-evolve during some years, there is still no consensus about their linkage. The author of [26] investigated the interaction of the Niño 4 index on the Indian Ocean SST and also the IOD index on the Pacific Ocean SST. In his study, the patterns of information flow from the Niño 4 index to Indian Ocean SST revealed an Indian Ocean dipole-like structure over the Indian Ocean basin. Furthermore, an ENSO-type pattern is also obtained over the Pacific Basin when the IOD index is applied to the Pacific Ocean SST. In our study, we applied the linear and nonlinear estimators to robustly measure the interaction mechanism between ENSO and IOD.

Here, the IOD and ENSO relationship was investigated with all the information exchange measures discussed above. The monthly time series of the Niño 4 index and Indian ocean dipole index were obtained from NOAA ESRL and JAMSTEC, respectively, for the period of 1958–2010 (i.e., 633 months). The lag-1 autocorrelation of Niño 4 is 0.8 and an IOD index of magnitude 0.9. Hence, a time series length longer than 500 time units is recommended to apply the methods mentioned in the previous examples. As the available length of the indices is 633 months, it is expected that information exchange detection and quantification could be robustly estimated by the information exchange methods mentioned in Section 2. However, detecting interaction delays might need a greater amount of time series while estimating TE [65]. Hence, we cross-checked if our interaction delays are physically consistent with the existing literature. As data input, the SST reanalysis named COBE-SST2 Sea Surface Temperature and Ice [66] was obtained from NOAA for the same period. Before applying the information exchange methods, the indices were deseasonalized, and the linear trend was removed to fulfill the stationarity criteria for TE [30] and IF-linear estimations.

Table 1 gives the information exchange between the two indices as quantified by the various methods. A permutation technique, under which the surrogates preserve the tuples $p(x_{n+1},x_{n}^{k},y_{n}^{l})$ for TE estimation is used to determine the significant information exchange within 95% confidence interval (100 samples). For more details into the technique, refer to [40]. The significance test for IF-linear follows [26]. Moreover, based on the AI, we chose the destination embedding k=1 due to the minimal increase from time lag 1 to time lag 10 months for both IOD and Niño 4 , while we scanned for the time interaction delay at which maximum information is exchanged from the source to the destination [31]. The parametric methods show significant information exchange from the Niño 4 index to IOD at a lag of 2 to 3 months, while significant information is exchanged from IOD to the Niño 4 index with a time lag of 0 and between 10 and 14 months. The instantaneous relation between the IOD and Niño 4 has been observed throughout the historical records. However, it is interesting to note that significant instantaneous information is exchanged from IOD to the Niño 4 index and not vice versa. This relation could be attributed to the feedback of the IOD on the Pacific Ocean due to the wind anomalies induced by ENSO over the Indian Ocean [59]. The time lagged information exchange results suggest that ENSO events tend to influence the IOD [67], and then the induced IOD tends to provide feedback to the ENSO [64,68]. The nonparametric TE-kernel and TE-kraskov estimators also exhibit a similar behavior except the TE-binning estimator. The free parameters are tuned and tested rigorously for numerical consistency.

**Figure 6:**
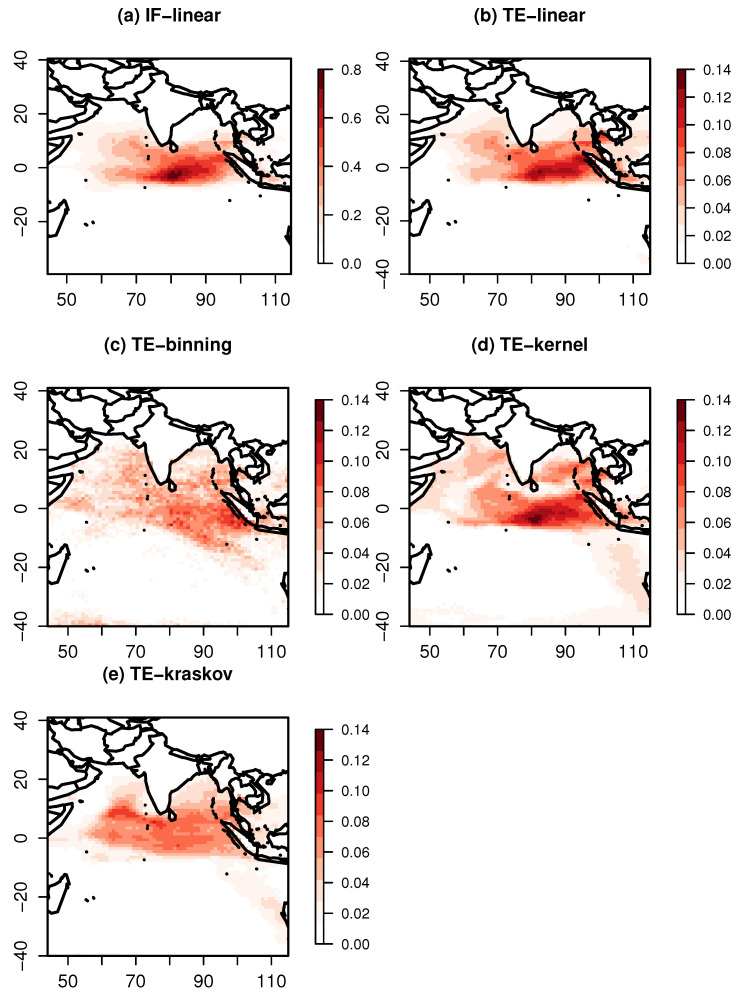
Information exchange from the Niño 4 Index to the Indian Ocean sea surface temperatures for the period of 1958–2010 measured by (**a**) the IF-linear method (nats/time) and (**b**–**e**) with different variants of the TE measure (in nats $\times 10^{-1}$).

We further investigated the patterns over the respective oceans at the time of maximum influence of the indices. Figure 6 shows significant information exchange from the Niño 4 index to the Indian Ocean SST at a lag of 3 months. The IF-linear shows the influence of Niño 4 on the Indian Ocean, especially on the Southeastern Indian Ocean, suggesting an information exchange from the Pacific Ocean to the Indian Ocean. The TE-linear also shows a significant exchange of information from the Pacific Ocean to the Indian Ocean with a maximum value of TE-linear near the Southeast Indian Ocean. While the TE-kernel also replicatef a similar pattern, the TE-binning estimator produced a spurious pattern. The results were also checked for consistency with various kernel widths ranging between 0.25 and 2. The TE-binning estimator could not reveal a similar pattern with various bin widths. The TE-kraskov estimation also revealed an information exchange from the Pacific Ocean to the Indian Ocean and was consistent within the range between 20 and 60 nearest neighbors.

**Figure 7:**
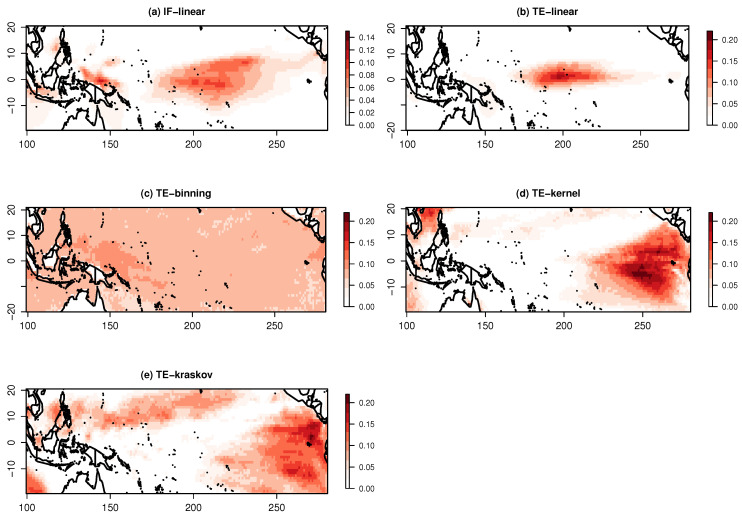
Information exchange from the Indian Ocean dipole Index to the Pacific Ocean sea surface temperatures for the period of 1958–2010 measured by (**a**) the IF-linear method (nats/time) and (**b**–**e**) with different variants of the TE measure (in nats $\times 10^{-1}$).

The significant information exchange pattern from IOD to the Pacific Ocean at a lag of six is represented in Figure 7. The IF-linear shows an information exchange over the central Pacific Ocean, suggesting an information exchange from the Indian Ocean to the Pacific Ocean. The TE-linear also shows that information is exchanged from the Indian Ocean to the Pacific Ocean with maximum values of TE occurring near the central Pacific Ocean. The TE-kernel and TE-kraskov estimators show nonlinear interactions near the East Pacific with numerical consistency from 0.25 to 2 kernel widths and 20 to 60 nearest neighbors, respectively, while the TE-binning produce spurious patterns without any numerical consistency for all varying bin widths. These nonlinear interactions need further investigation.

As the current example is a large-scale process and the variables are expected to be near-Gaussian, and as all the estimators (except TE-binning) showed a bidirectional information exchange, it could be concluded that a bidirectional information exchange exists between the Pacific and Indian Ocean SST. However, as mentioned earlier, we limited our investigation to two-dimensional systems, here between ENSO and IOD. In real-world climate systems, there could always be an unobserved influence of another system on the information exchange estimations. Hence, care is to be taken before drawing any conclusions; furthermore, the information exchange magnitudes are quite smaller compared with the idealized test cases.

#### 3.2.2. Information Exchange between Nao and European Near-Surface Temperatures

The North Atlantic Oscillation (NAO) is one of the dominant modes in the climate system which influences the European climate and many parts of the Northern Atlantic [69]. The NAO characterizes the strength of the subtropical high and polar low. The NAO is measured through the NAO index, which is obtained by the normalized pressure difference between the stations located in the Azores and Iceland [70]. A positive NAO represents a deeper low over Iceland and stronger subtropical high than normal and vice versa.

There have been several studies suggesting the influence of the NAO on the winter temperatures over Europe [71,72]. One of the physical mechanisms in which the NAO influences the temperatures is by the advection of the heat by anomalous mean flow [71]. Another mechanism through which the temperatures are affected is through modulation of radiation by the cloud cover [72] through altering the storm track directions. In the current study, we investigated the information exchange from NAO to winter temperatures over Europe and checked if all the methods reproduce this relation.

The NAO index and the near-surface air temperatures (*T*) were obtained from the CRU database [73] held at the British Atmospheric Data Centre, RAL, UK for the period of 1901 to 2016. December, January, and February temperatures were chosen as the winter months. The total time series length of 348 months was considered. The autocorrelation of NAO is of magnitude 0.1 with a time lag of 1 month, and surface air temperature autocorrelation is about 0.05 with a time lag of 1 month. Because of the low autocorrelations, we expected the methods mentioned in Section 2 to be robust with given time series length. Moreover, the AI for *T* and NAO showed a minimal increase from time lag 1 to time lag 10, and hence, k=1 was chosen, while we scanned for an interaction delay at which the source exchanges maximum information exchange. The PRUDENCE regions [74] over Europe were selected, and spatial mean air temperatures over different regions were used to detect the information exchange.

Table 2 shows the information exchange between the NAO and *T* over two PRUDENCE regions (the British Isles and Scandinavia). The parametric IF-linear shows significant information exchange from *T* over the British Isles to the NAO index at an interaction delay of zero days. The TE estimators show a significant information exchange bidirectionally between NAO and *T* over the British Isles except for the TE-binning estimator. For Scandinavia, IF-linear shows information exchange from NAO to *T*, while TE estimators show bidirectional information exchange. Over other PRUDENCE regions, similar bidirectional information exchange ess observed except for the Mediterranean region. These results seem to be implausible, as one would not expect any information exchange from *T* over Europe to the NAO index. This unrealistic estimation of information exchange could have arisen from a common influence by a hidden third variable. Earlier studies have observed the sensitivity of TE estimation to a hidden variable [75]. From previous literature, a possible influence on NAO might arise from the variations in sea surface temperatures, sea ice, volcanic activity, and solar activity [76], which also influence the *T* over Europe.

## 4. Conclusions

This work targeted detecting and quantifying interactions in climate phenomena through asymmetric methods from information theory, IF, and TE. However, due to the difficulty in their estimations, we initially tested various estimators of these methods to idealized systems and then to two important climate phenomena. We limited our discussions only to two-dimensional systems.

The parametric estimators assuming linearity, such as the rigorously derived IF-linear and axiomatically proposed TE-linear, detected and reliably quantified the unidirectional and bidirectional information exchange in the idealized linear systems. IF-linear was able to detect the unidirectional information exchange for the tested unidirectional nonlinear system, whereas the TE-linear failed to do so. For the bidirectional nonlinear Heńon maps, both linear estimators failed to detect and quantify the information exchange. Hence, care has to be taken if linear information exchange measures is applied in climate system diagnosis, especially if the system variables have non-Gaussian distributions. However, these two estimators, IF-linear and TE-linear, were robust and reliable for the discussed linear systems and, in addition, IF-linear also for a weakly nonlinear system. For all the idealized systems discussed here, the nonlinear implementation of IF might reveal the interactions, but since we focused on climate applications given time series with an unknown dynamical model, we used IF-linear, which does not require system dynamics.

Among the nonparametric estimators, the TE-binning failed to be useful as a robust estimator. Even though the TE-kernel and TE-kraskov passed the idealized tests, their implementations had to be tuned to get consistent numerical results. Slow convergence of information exchange with the TE-kraskov estimator with increase in time series length was also noted. Therefore, we concluded that both reliable nonparametric estimators should be jointly applied and their implementation should be optimized for consistent results before any quantitative interpretation of the investigated nonlinear system is drawn. This conclusion is conditioned on the availability of long enough data time series. The composite use of TE-kernel and TE-kraskov showed that the dynamics of the Lorenz-96 model is dominated by the slow subsystem.

For real climate applications, i.e., information exchange between the Indian and Pacific Oceans, the parametric and reliable nonparametric estimators showed a significant bidirectional information exchange. Moreover, the time lag of significant information exchange from Pacific to the Indian Ocean was about 2 to 3 months. An instantaneous information exchange from the Indian to Pacific Ocean was detected and also with a time lag of about 10 to 12 months. The respective spatial patterns over the Indian and Pacific Oceans revealed a significant bidirectional information exchange. Hence, given the consistent estimations, we concludef that a bidirectional information exchange exists between the Pacific and Indian Oceans, as expected from literature [64,67,68]. However, given the limitations of TE and IF-linear, a possibility of a hidden influence by another system cannot be ruled out. This requires further analysis.

For the relation of NAO and European winter air temperatures, the estimators showed significant bidirectional information exchange. The process mechanism from NAO to European temperature is often discussed in the literature [71,72]. However, the measured information exchange from European temperatures to the NAO cannot be explained by a straightforward process chain. This indicates an influence from a third hidden variable as a common driver.

Thus, even though TE and IF-linear are useful measures which allow for quantification of interactions and their directionality, their limitations and the system at hand need to be taken into account carefully before drawing any conclusions from their estimations. Hence, we propose a composite use of the information theory methods with parameter testing for various applications, for example, as a robust model evaluation framework. While this study was limited in investigating the relationship between two systems, in the future study, the authors plan to investigate interaction measures based on information theory in higher-dimensional climate system networks.

## Figures and Tables

**Table 1:** Information exchange between the Niño 4 (N4) and the Indian Ocean dipole (IOD) index (* refers to significant information exchange).

Method	N4 to IOD (lag=3)	IOD to N4 (lag=0)	IOD to N4 (lag=7)	Units	
IF-linear	1.0 *	1.2 *	1.1 *	nats/month	$\times 10^{-2}$
TE-linear	0.7 *	1.3 *	1.4 *	nats	$\times 10^{-2}$
TE-binning	0.5 *	0.9 *	0.7	nats	$\times 10^{-2}$
TE-kernel	0.3 *	1.5 *	1.6 *	nats	$\times 10^{-2}$
TE-kraskov	0.1 *	1.1 *	0.9 *	nats	$\times 10^{-2}$

**Table 2:** Information exchange between North Atlantic Oscillation (NAO) and winter near-surface temperatures (* refers to significant information exchange).

Method	NAO to TS	TS to NAO	Units	Region
IF-linear	0.02	0.09 *	nats/month	British Isles
TE-linear	0.318 *	0.314 *	nats	British Isles
TE-kernel	0.4 *	0.38 *	nats	British Isles
TE-kraskov	0.3 *	0.2 *	nats	British Isles
IF-linear	0.08 *	0.05	nats/month	Scandinavia
TE-linear	0.14 *	0.14 *	nats	Scandinavia
TE-kernel	0.24 *	0.2 *	nats	Scandinavia
TE-kraskov	0.16 *	0.17 *	nats	Scandinavia

## Abbreviations

The following abbreviations are used in this manuscript:

TE	Transfer Entropy
IF	Information Flow
MI	Mutual Information
CMI	Conditional Mutual Information
AI	Active Information storage
C	Coupling Coefficient
NAO	North Atlantic Oscillation
ENSO	El-Niño Southern Oscillation
SST	Sea Surface Temperature
IOD	Indian Ocean dipole
